# Long non-coding RNA metastasis associated in lung adenocarcinoma transcript 1 (MALAT1) interacts with estrogen receptor and predicted poor survival in breast cancer

**DOI:** 10.18632/oncotarget.9364

**Published:** 2016-05-13

**Authors:** Nai-si Huang, Ya-yun Chi, Jing-yan Xue, Meng-ying Liu, Sheng Huang, Miao Mo, Shu-ling Zhou, Jiong Wu

**Affiliations:** ^1^ Department of Breast Surgery, Fudan University Shanghai Cancer Center, China; ^2^ Department of Oncology, Shanghai Medical College, Fudan University, China; ^3^ Department of Clinical Statistics, Fudan University Shanghai Cancer Center, China; ^4^ Department of Pathology, Fudan University Shanghai Cancer Center, China; ^5^ Collaborative Innovation Center of Cancer Medicine, China

**Keywords:** MALAT1, long non-coding RNA, breast cancer, estrogen receptor

## Abstract

Metastasis associated in lung adenocarcinoma transcript 1 (MALAT1), a lncRNA that was first recognized as a prognostic parameter for patient survival of stage I lung cancer, is up-regulated in multiple human malignancies, including breast cancer. However, the mechanism of its function remained elusive. In the current study, by examining MALAT1 expression on mRNA level, we demonstrated that compared with MCF10A, MALAT1 expression was up-regulated in the majority of breast cancer cell lines (9/12). In 26 pairs of estrogen receptor (ER)-positive breast cancer samples, MALAT1 expression was significantly up-regulated compared with adjacent normal tissues (*P* = 0.012). Furthermore, of 204 breast cancer patients, high MALAT1 expression was associated with positive ER (*P* = 0.023) and progesterone receptor (PR) (*P* = 0.024) status. Further analysis using TCGA database revealed that ER and its target genes *PGR* and *CCND1*, were overexpressed in *MALAT1* altered group compared with unaltered group, both on the mRNA and protein level. Lastly, we verified MALAT1's prognostic value in breast cancer. At the cut-off value of 75%, MALAT1 was the only independent prognostic factor of recurrence-free survival (RFS) in ER-negative patients in a multivariate Cox regression model (hazard ratio [HR] = 2.83, 95% confidence interval [CI] 1.02–7.83). MALAT1 overexpression was also associated with poor RFS in tamoxifen treated ER-positive breast cancer patients, which might serve as a potential biomarker to predict endocrine treatment sensitivity.

## INTRODUCTION

Noncoding RNA (ncRNAs) is represented by approximately 97% of transcribed RNA molecules, and only 3% of the RNAs are protein-coding messenger RNAs [1]. Long intergenic non-coding RNAs (lncRNAs), which can range from several hundred to tens of thousands of bases, are another class of newly discovered ncRNAs. LncRNAs control transcriptional alteration, implying that the difference in lncRNA profiling between normal and cancer cells is not merely a secondary effect of cancer transformation and that lncRNAs are strongly associated with cancer progression [2, 3].

LncRNA metastasis-associated in lung adenocarcinoma transcript 1 (MALAT1), was first recognized as a prognostic parameter for patient survival of stage I lung adenocarcinoma or squamous cell carcinoma patients in 2003 [4]. MALAT1 is also associated with several human tumor entities, including liver cancer, renal cell carcinoma, bladder carcinoma, colorectal cancer and cervical cancer, and it is generally regarded as a negative predictor of tumor prognosis [5, 6]. MALAT1 facilitates cell growth, migration, and invasion in malignancies [7–9]; however, the mechanism underlying these effects has remained elusive [10].

MALAT1 is also an abundantly expressed lncRNA in primary breast cancer [12]. Mutations and deletions in the human MALAT1 gene were recently discovered in luminal breast cancer [13, 14]. Compared with normal breast tissue, MALAT1 expression was up-regulated significantly in primary breast cancer and lymph node metastasis [15]. A recent study demonstrated that, down-regulation of MALAT1 mouse mammary carcinoma model resulted in slower tumor growth accompanied by significant differentiation into cystic tumors and a reduction in metastasis [16]. Nevertheless, little is known about the mechanism through which MALAT1 exerts its oncogenic activity in breast cancer, as well as its interaction with other molecules. The aim of the current study is to explore the role of MALAT1 in breast cancer oncogenesis and its potential value as a prognostic biomarker.

## RESULTS

### MALAT1 expression profile in breast cancer cell lines

**Figure 1:**
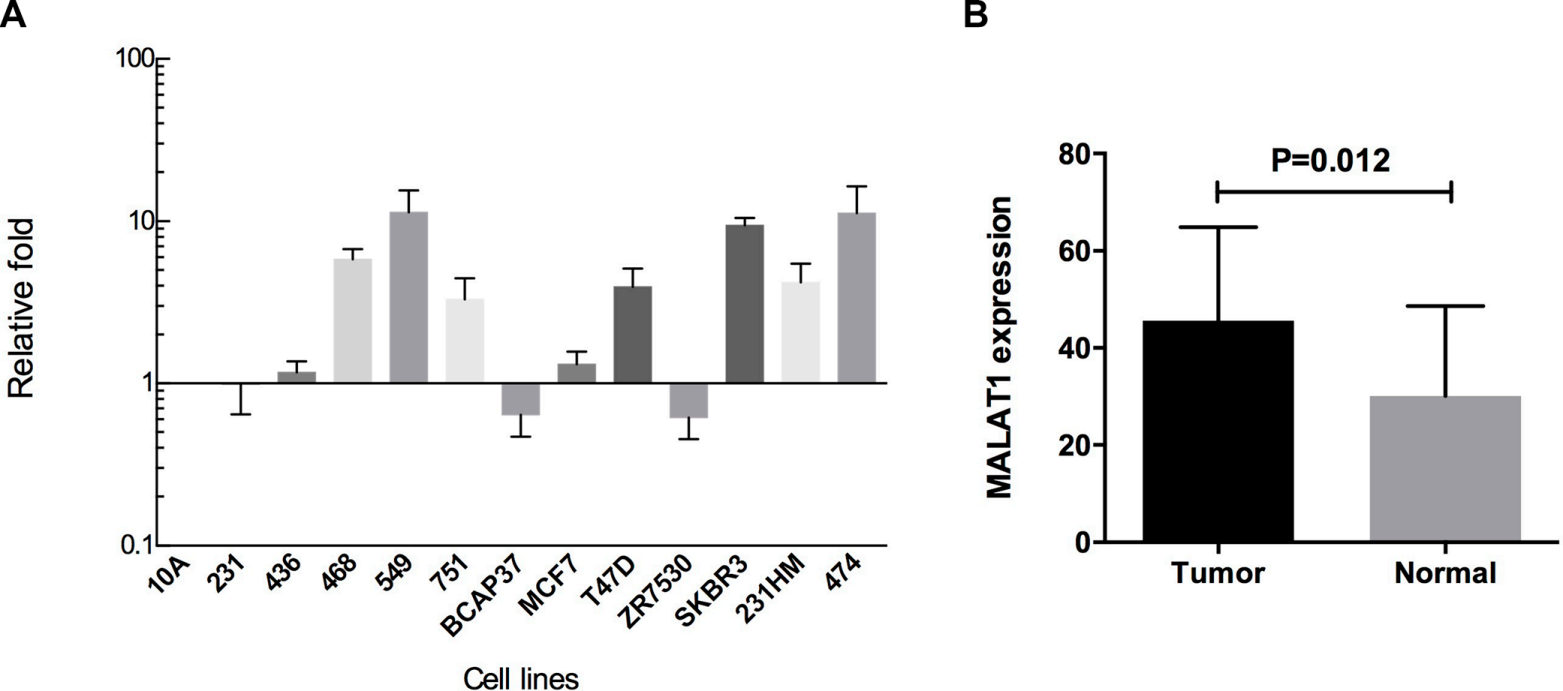
(**A**) MALAT1 expression profile in 13 breast cell lines. Compared with the normal breast cell line MCF10A, MALAT1 expression was up-regulated in MDAMB436, MDAMB468, BT549, ZR751, MCF7, T47D, SKBR3, MDAMB231HM, and BT474; (**B**) MALAT1 expression was significantly up-regulated compared with adjacent normal tissues (*P* = 0.012) in ER-positive breast cancers.

Expression profiles for MALAT1 were examined in 12 breast cancer cell lines. As shown in Figure 1A, compared with normal breast cell line MCF10A, MALAT1 expression was up-regulated in most breast cancer cells (9/12, 75.0%), including MDAMB436, MDAMB468, BT549, ZR751, MCF7, T47D, SKBR3, MDAMB231HM, and BT474.

### MALAT1 was overexpressed in ER-positive breast cancer tissues

Then, we detected the expression of MALAT1 in 33 pairs of breast cancer tissues and their adjacent normal tissues. The expression of MALAT1 tended to be higher in breast cancer tissues than in normal tissue but was not significantly different between the two (*P* = 0.075). Among 33 pairs of breast cancer, 26 pairs were ER positive. MALAT1 expression was significantly up-regulated compared with adjacent normal tissues (*P* = 0.012) in ER-positive breast cancers (Figure 1B).

### Correlations between MALAT1 expression and ER in breast cancer patients

To identify the clinical relevance of MALAT1 expression in breast cancer, the correlation between MALAT1 expression and tumor clinical-pathological parameters was analyzed in 204 breast cancer tissues. The expression levels of MALAT1 in breast cancer were categorized as low or high expression at the cut-off value of the median. The correlation between MALAT1 expression levels and patients' clinical-pathological characteristics was summarized in Table 1. High MALAT1 expression was associated with positive ER (*P* = 0.023) and PR (*P* = 0.024) status, as well as lower tumor grades (*P* = 0.025). Nevertheless, when stratified ER status, MALAT1 expression was not correlated with tumor grade in ER-positive group (*P* = 0.417) or ER-negative group (*P* = 0.055).

**Table 1:** Relationship between MALAT1 expression and clinical-pathological characteristics of breast cancer patients

Characteristics	*N*	MALAT1 expression		χ2	*P*-value
		Low	High		
Age (years)					
≤ 50	98	49 (48.0%)	49 (48.0%)	0.000	1.000
> 50	106	53 (52.0%)	53 (52.0%)		
Menopausal status					
Pre	91	44 (41.3%)	47 (46.1%)	0.179	0.778
Post	113	58 (56.9%)	55 (53.9%)		
Tumor size (cm)^*^					
≤ 2	74	33 (32.4%)	41 (40.2%)	1.357	0.308
> 2	130	69 (67.6%)	61 (59.8%)		
Lymph node status					
Negative	109	56 (54.9%)	53 (52.0%)	0.177	0.779
Positive	95	46 (45.1%)	49 (48.0%)		
Tumor grade					
I–II	103	45 (47.9%)	58 (65.2%)	5.558	**0.025**
III	80	49 (52.1%)	31 (34.8%)		
ER status					
Negative	85	51 (50.0%)	34 (33.7%)	5.565	**0.023**
Positive	118	51 (50.0%)	67 (66.3%)		
PR status					
Negative	91	54 (52.9%)	37 (36.3%)	5.733	**0.024**
Positive	113	48 (47.1%)	65 (63.7%)		
HER-2 status					
Negative	106	57 (61.3%)	49 (57.0%)	0.344	0.648
Positive	73	36 (38.7%)	37 (43.0%)		
LVI					
Negative	122	65 (66.3%)	57 (58.8%)	1.191	0.302
Positive	73	33 (33.7%)	40 (41.2%)		

* Only the size of invasive tumor is included.

ER: estrogen receptor; PR: progesterone receptor; HER-2: human epidermal growth factor receptor-2; LVI: lymph vascular invasion.

**Figure 2:**
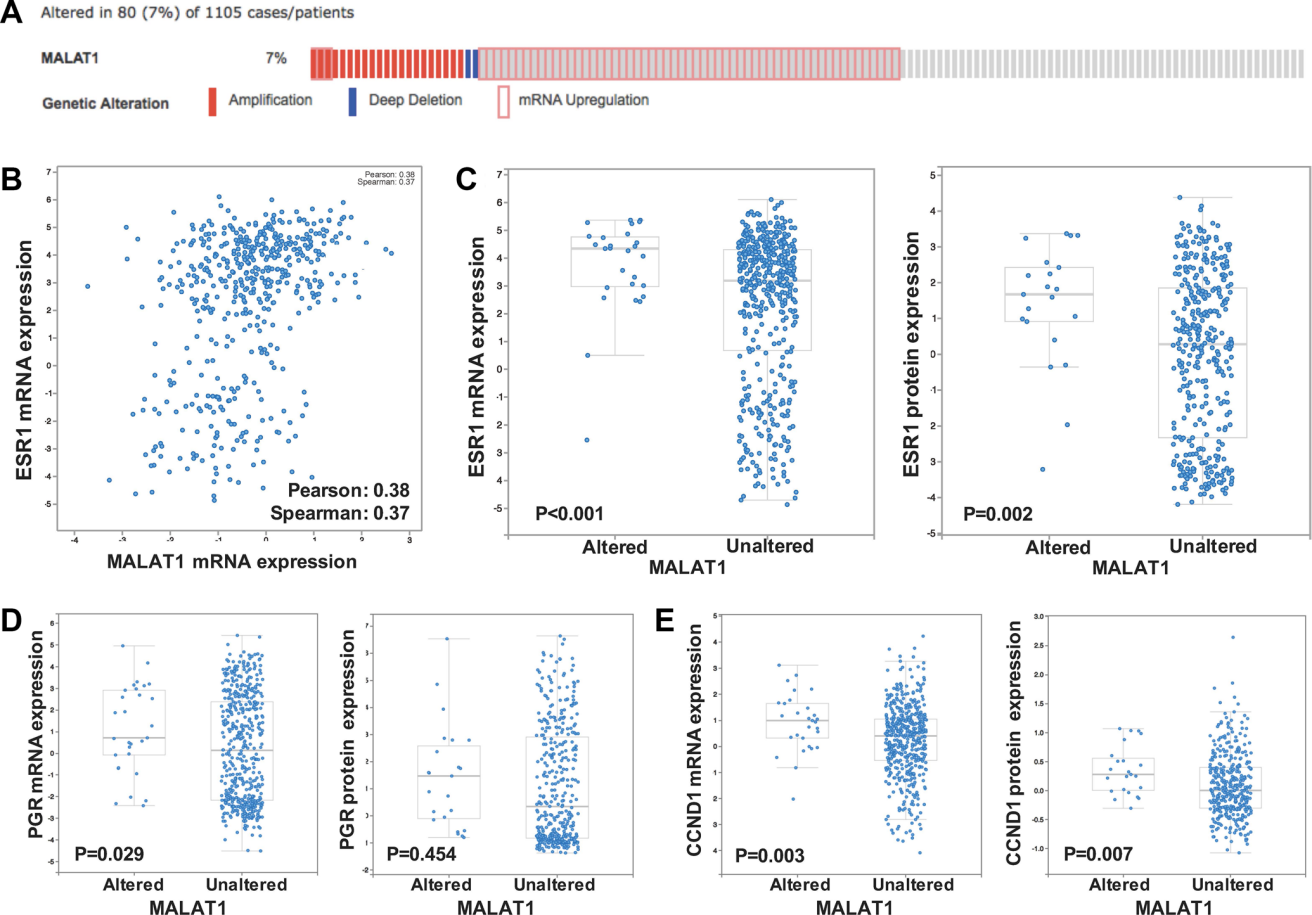
MALAT1 correlated with ER and its downstream genes' expression (**A**) MALAT1 was amplified or up-regulated in 7% of breast cancer cases; (**B**) MALAT1 and ER expression was moderately correlated on the mRNA level; (**C**) MALAT1 was related to ER expression on the mRNA and protein level; (**D**) and (**E**), MALAT1 was related to the expression of ER's target genes, *PGR* and *CCND1*, on the mRNA and protein level.

Then we used the TCGA [17, 18] database to testify our observation and came to similar conclusions. MALAT1 was amplified or up-regulated in 7% of breast cancer cases (Figure 2A). ER expression was moderately correlated on mRNA level (Pearson = 0.38, Spearman = 0.37). Further analysis revealed that, ER was up-regulated in *MALAT1* altered group compared with unaltered group, both on the mRNA level (*P* < 0.001) and protein level (*P* = 0.002). ER's target genes, *PGR* and *CCND1* were also overexpressed in *MALAT1* altered group, indicating MALAT1 might play a role the regulation of ER expression in breast cancer (Figure 2B–2E).

### The prognostic role of MALAT1 in breast cancer

**Figure 3:**
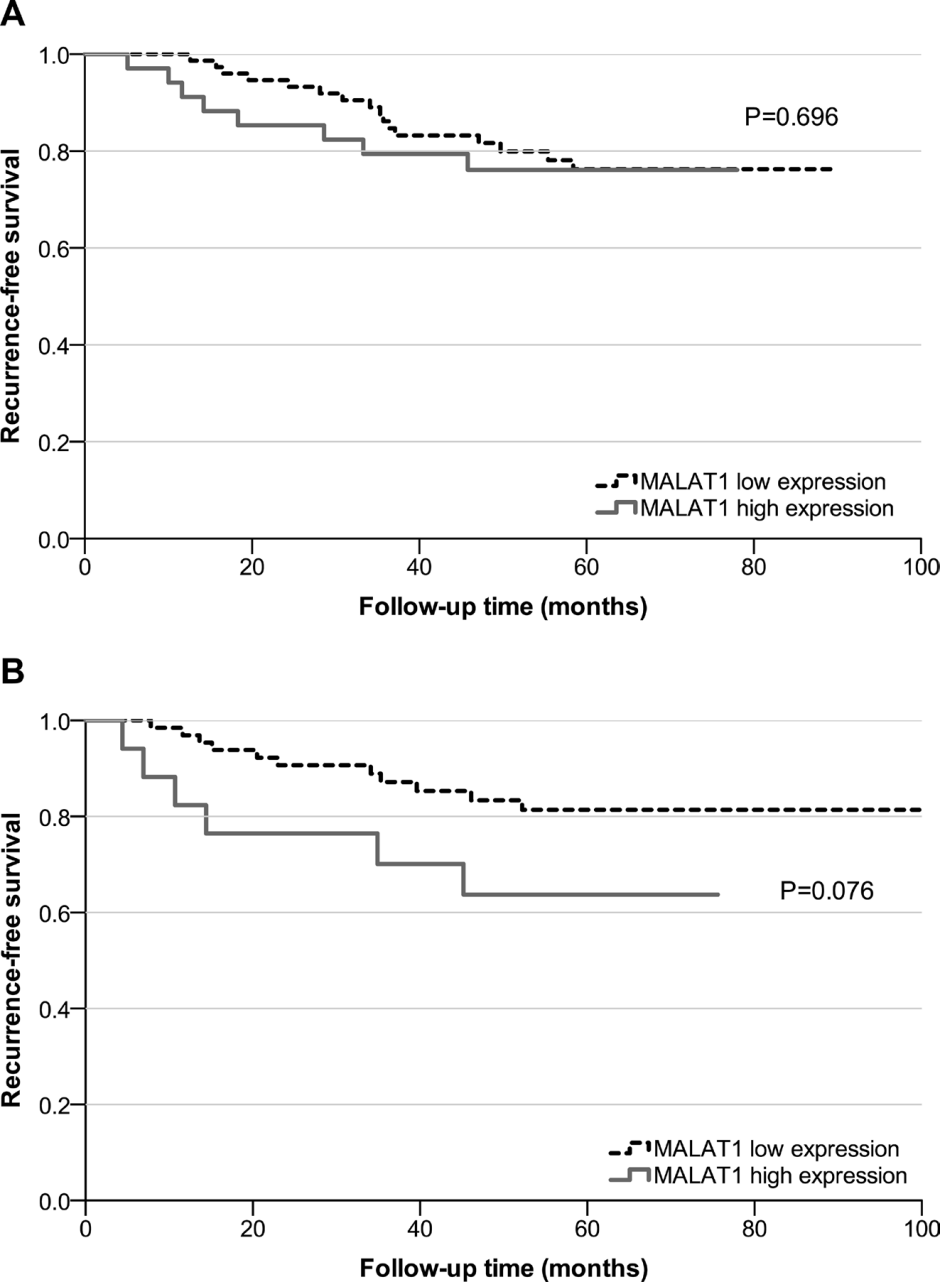
Survival analysis in breast cancer patients based on MALAT1 expression (**A**) Recurrence-free survival based on low MALAT1 expression versus high MALAT1 expression in the ER-positive group; (**B**) Recurrence-free survival based on low MALAT1 expression versus high MALAT1 expression in the ER-negative group.

In 204 breast cancer patients, the median follow-up time was 65.0 months (IQR: 47.0–72.2). 41 cases (20.9%) developed recurrence, and 22 patients (11.2%) died (18 patients died of breast cancer, three patients died of other malignant tumors, and one patient died of heart disease). At the cut-off value of 75% of MALAT1's expression, in the ER-positive group, the 5-year RFS rates of the low expression group versus the high expression group were 76.3% and 76.1%, respectively (*P* = 0.696; Figure 3A). In the ER-negative group, the 5-year RFS rates were 81.4% and 63.7%, respectively (*P* = 0.076; Figure 3B). No difference was detected in OS. MALAT1 expression level, tumor size, lymph node status, and lymphovascular invasion (LVI) status were included in a multivariate Cox regression analysis. As shown in Table 2, MALAT1 expression level was the only independent prognostic factor of poor RFS in the ER-negative patients; however, it did not have prognostic value in the ER-positive subset of patients.

**Table 2:** Multivariate Cox regression analysis of prognostic factors for RFS in breast cancer patients according to ER status

Variables	ER-positive			ER-negative		
	HR	95% CI	*P*-value	HR	95% CI	*P*-value
MALAT-1 expression high vs. low	1.54	0.62–3.80	0.355	2.83	1.02–7.83	**0.045**
Lymph node status positive vs. negative	4.43	1.15–17.05	**0.030**	0.98	0.29–3.33	0.973
Tumor size ≤ 2 cm vs. > 2 cm	4.18	0.94–18.54	**0.060**	2.23	0.61–6.75	0.250
LVI positive vs. negative	0.87	0.32–2.35	0.785	2.99	0.91–9.80	0.071

ER: estrogen receptor; CI: confidential interval; HR: hazard ratio; LVI: lymph vascular invasion.

**Figure 4:**
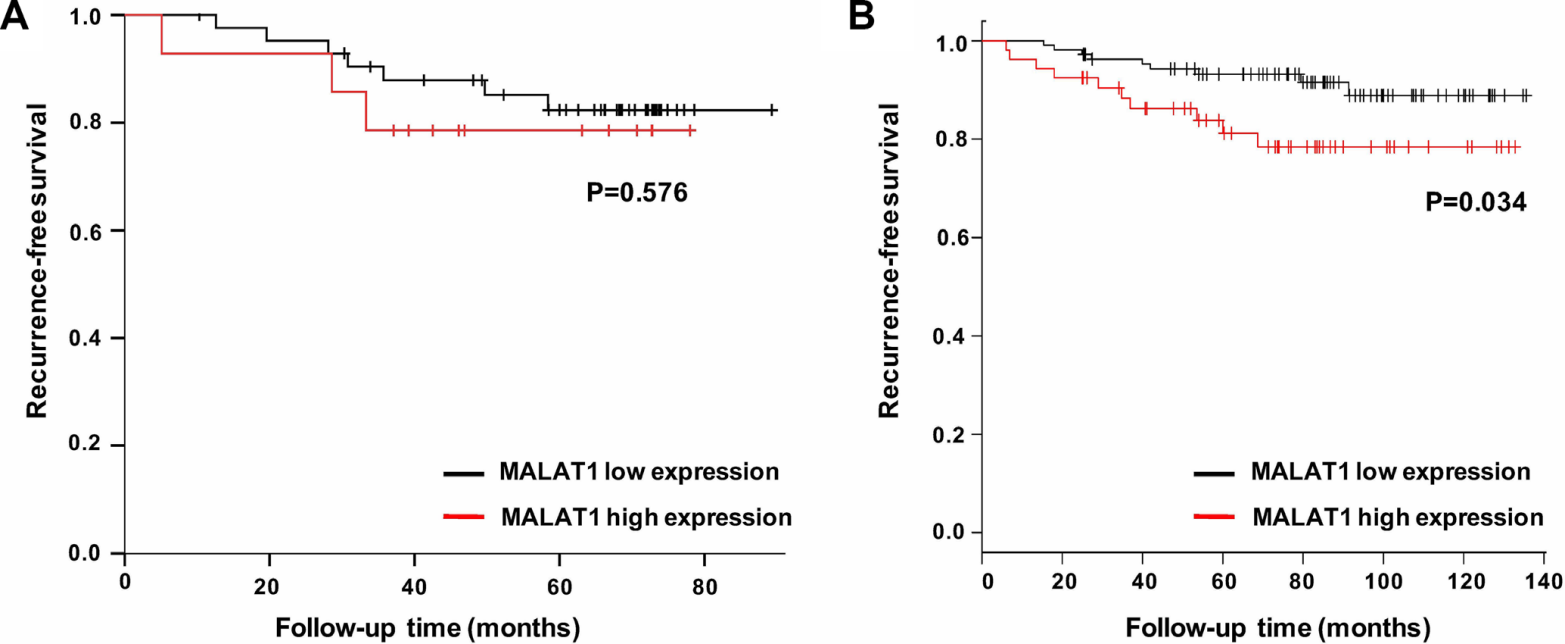
Survival analysis in tamoxifen treated ER-positive breast cancer patients based on MALAT1 expression (**A**) Recurrence-free survival based on low MALAT1 expression versus high MALAT1 expression in a cohort of 57 patients in our institute; (**B**) Recurrence-free survival based on low MALAT1 expression versus high MALAT1 expression in Gyorffy's dataset of 161 patients.

Next, we limit the cohort to 57 tamoxifen treated ER-positive breast cancer patients and re-analyzed our data. Although no statistically significance was detected due to small sample size and few recurrences, the RFS rates for MALAT1 high expression versus low expression group were 78.6% and 83.7% (*P* = 0.576), respectively (Figure 4A). Then we used Gyorffy's dataset [19] to conduct survival analysis and found that, in 161 tamoxifen treated ER-positive patients, high MALAT1 expression level predicted poor RFS (HR = 2.56, 95%CI: 1.04–6, *P* = 0.034) at the cut-off value of 75%, which was consistent with the result of our cohort (Figure 4B).

## DISCUSSION

Accumulating evidence has shown that lncRNAs play a critical biological role in cellular development and human diseases [20–22]. MALAT1, also known as nuclear-enriched abundant transcript 2 (NEAT2), is highly expressed in mammalian species [23]. Many of MALAT1's vital functions are related to nuclear processes, such as gene splicing, gene expression and nuclear organization [14]. In multiple human cancers, MALAT1 plays a role in carcinogenesis, including tumor cell proliferation, apoptosis, migration and metastasis [24–26]. In the present study, our results indicated that MALAT1 expression was up-regulated in multiple breast cancer cell lines compared with normal breast cell line, as well as in ER-positive breast cancer tissues compared with adjacent normal breast cancer tissues. These data correlated with previous studies indicating that MALAT1 was overexpressed in primary breast cancer and promoted proliferation of breast cancer cells [27–29]. We identified for the first time that MALAT1 expression was correlated with ER and its target genes in breast cancer, providing a new perspective in MALAT1 function. The mechanism through which MALAT1 regulates ER expression remained to be explored. Arun et al. reported in their study that *ESR1*, coding for ER, was found to be alternatively spliced in its 5′ untranslated region (UTR) in MALAT1 knock-down samples; however, it remains to be further investigated whether the alternative splicing of *ESR1* confers the stability of ER [16].

The prognostic value of MALAT1 in breast cancer was controversial in our study. High MALAT1 expression was an independent predictor of poor RFS in ER-negative patients, while not in ER-positive patients. Possible reasons include patients receiving various endocrine therapies that potentially affected MALAT1 expression and its predictive role; and relative short follow-up time. Several signaling pathways have been proposed to explain the oncogenesis of MALAT1 in breast cancer. Jin et al. demonstrated a reciprocal negative control relationship between MALAT1 and miR-1; MALAT1 might exert its function through the miR-1/slug axis [27]. Bamodu et al. identified MALAT1 as a mediator of KDM5B oncogenic potential in triple-negative breast cancer, and could be reversed by hsa-miR-448 [30]. Furthermore, MALAT1 reversed the inhibitory effect of miR-124 on breast cancer proliferation and was involved in the cyclin-dependent kinase 4 (CDK4) expression [29].

Most interestingly, we uncovered that high MALAT1 expression level was associated with tamoxifen treatment failure in ER-positive breast cancer. As MALAT1 might play a role in regulating ER expression [16], it is possible that MALAT1 affects tamoxifen resistance in an ER- dependent manner. Previous studies also demonstrated relevant variation in MALAT1 transcript abundance in tamoxifen-treated and untreated ER-positive breast cancer [12, 31, 32]. Taken together, MALAT1 might induce tamoxifen resistance via regulating transcription and splicing of *ESR1* and affecting ER signaling. MALAT1 may provide a new perspective in tamoxifen resistance mechanism and help to develop new treatment target for ER-positive breast cancer. Meanwhile, MALAT1 could serve as a potential biomarker for endocrine therapy sensitivity, which is essential for personalized treatment in these patients.

## MATERIALS AND METHODS

### Cell line and regents

Twelve breast cell lines and one normal breast cell line (MCF10A) were obtained from the cell bank of our lab. ZR751, ZR7530, MCF7, SKBR3, BT474 and T47D cells were cultured in RMPI1640 medium. MDAMB231 and MDAMB231HM cells were cultured in F15 medium. MDAMB436, MDAMB468, and BT549 were cultured in DMEM medium. MCF10A was cultured in F12/DMEM 1:1 medium. All cells were cultured with 10% fetal bovine serum (FBS), 100 units/ml penicillin, and 100 ug/ml streptomycin at 37°C and 5% CO_2_. The pathological features of 13 cell lines are listed in Supplementary Table 1.

### Patient samples

Our study included two cohorts of patients. Firstly, 33 pairs of primary non-metastatic breast tumors and their matched adjacent normal tissues were obtained. Next, breast cancer tissues of 204 patients treated in our clinic between 2007 and 2009 were included. The following exclusion criteria were applied: (1) metastatic breast cancer, (2) patients who received neo-adjuvant chemotherapy prior to surgery and (3) patients who were diagnosed with recurrent breast cancer upon surgery. All breast samples were obtained and frozen in liquid nitrogen immediately after surgery and stored in −80°C freezers in the tissue bank in Fudan University Shanghai Cancer Center (FUSCC) until RNA extraction. The percentage of cancer components exceeded 90% in each breast cancer sample. Pathology diagnosis, ER, PR, and HER2 status were reviewed by academic pathologists according to the World Health Organization (WHO) classification and American Society for Clinical Oncology (ASCO) guidelines. Patient baseline characteristics and follow-up information were included in the final analysis. Informed consent was obtained from all subjects in the current study. The Ethical Committee of FUSCC for Clinical Research approved the protocol of the study.

### RNA extraction and quantitative RT-PCR

Total RNA was extracted from clinical specimens and cell lines using TRIzol reagent (Invitrogen) following the manufacturer's protocol. After converting total RNA to cDNA in a reverse transcription (RT) reaction, cDNA templates were mixed with gene-specific primers for MATAT-1 (forward primer AAAGCAAGGTCTCCCCACAAG, reverse primer GGTCTGTGCTAGATCAAAAGGCA) and internal control GAPDH. Quantitative real time polymerase chain reaction (qRT-PCR) was used to quantitate MALAT1 expression. qRT-PCR was performed with the ABI7900 system. Melting curve analysis was used to monitor the specificity of the PCR products. The PCR for each sample was performed in triplicate. In addition, 2-delta Ct values were used to determine relative expression.

### Statistical and bioinformatics analysis

A paired Wilcoxon signed rank test was used to examine MALAT1 expression in breast cancer tissues versus adjuvant normal tissues. A Pearson's χ^2^ test was performed to detect the correlation between MALAT1 expression and breast cancer clinical-pathological characteristics. In survival analysis, we used a Kaplan-Meier and Cox proportional hazards model to examine whether MALAT1 expression impacted prognosis. Two-tailed *P*-values were adopted, and *P* < 0.05 was considered significant. All statistical analyses were performed using SPSS version 20.0 (SPSS Inc., Chicago, IL, USA) and SAS version 9.2 (SAS Institute Inc., Cary, NC, USA). Oncoprint analysis of the provisional breast cancer TCGA dataset were conducted via http://www.cbioportal.org. In the survival analysis of Gyorffy's dataset, the cut-off value of MALAT1 was set at 75% and only Tamoxifen treated ER-positive patients were included.

## SUPPLEMENTARY MATERIALS TABLE


