# Supplementary material for: Long non-coding RNA metastasis associated in lung adenocarcinoma transcript 1 (MALAT1) interacts with estrogen receptor and predicted poor survival in breast cancer
Source: Oncotarget. 2016 May 13;7(25):37957–65. doi: 10.18632/oncotarget.9364 (PMC5122363; doi:10.18632/oncotarget.9364)
Supplement: Supplementary file 1 [file oncotarget-07-37957-s001.pdf]

Long non-coding RNA metastasis associated in lung adenocarcinoma transcript 1 (MALAT1) interacts with estrogen receptor and predicted poor survival in breast cancer

Supplementary Materials

Supplementary Table S1: Pathological features of MCF10A and 12 breast cell lines

Cell line	Subtype	ER	PR	HER2	TP53	Tumor type	Cultural media
MCF10A	Basal[2,3,6];BasalB[8]	–[3,6,8]	–[3,6,8]	+/–[3];–[6]	WT[8]	F	F12/DMEM
BCAP37*	NR	–	NR	+	Mutant	MC	1640
BT474	Luminal[1,2,6,8];LuminalB[3,4]	–[3,5];+[6,8]	+[3,6,8];–[5]	+[3,5,6,8]	Mutant[5,7]	IDC	1640
BT549	Basal[2,6];BasalB[8]	–[5,6,8]	–[5,6,8]	–[5,6]	Mutant[5,7,8]	DC, pap	DMEM
MCF7	Luminal[1,2,6,8];LuminalA[3,4]	+[3,5,6,8]	+[3,5,6,8]	+/–[3];–[6]	WT[5,7,8]	IDC	1640
MDAMB231	Basal[2,3,6];BasalB[1,8]	–[3,5,6,8]	–[3,5,6,8]	+/–[3];–[5,6]	Mutant[5,7,8]	AC	F15
MDAMB-231HM*	Basal	–	–	–	Mutant	AC	F15
MDAMB436	BasalB[8]	–[8]	–[8]	NR	NR	IDC	DMEM
MDAMB468	Basal[2,3,4];BasalA[1,6,8]	–[6,8]	–[6,8]	–[6]	Mutant[5,7]	AC	DMEM
SKBR3	Luminal[1,2,6,8];HER2[3,4]	–[6,8]	–[6,8]	+[5,6]	WT[5]	AC	1640
T47D	Luminal[1,2,6,8];LuminalA[4]	+[5,6,8]	+[5,6,8]	–[6]	Mutant[5,8];WT[7]	IDC	1640
ZR751	Luminal[1,2,3,6,8]	+[3,5,6,8]	+[3,5];–[6,8]	+[3,5];–[6]	WT[5,7]	IDC	1640
ZR7530	Luminal[3,6,8]	+[6,8]	–[6,8]	+[5,6]	WT[5]	IDC	1640

*Subject to data in our lab.
ER/PR: positivity; HER2: over expression; TP53: WT, wild type protein; AC, adenocarcinoma; F, fibrocystic disease; IDC, invasive ductal carcinoma; Pap, papillary; MC, medullary carcinoma; NR: not reported.

REFERENCES

1. Battula VL, Shi Y, Evans KW, Wang RY, Spaeth EL, Jacamo RO, Guerra R, Sahin AA, Marini FC, Hortobagyi G, Mani SA, Andreeff M. Ganglioside GD2 identifies breast cancer stem cells and promotes tumorigenesis. J Clin Invest. 2012; 122: 2066–78.
2. Marotta LL, Almendro V, Marusyk A, Shipitsin M, Schemme J, Walker SR, Bloushtain-Qimron N, Kim JJ, Choudhury SA, Maruyama R, Wu Z, Gönen M, Mulvey LA, et al. The JAK2/STAT3 signaling pathway is required for growth of CD44+CD24– stem cell–like breast cancer cells in human tumors. J Clin Invest. 2011; 121:2723–35.
3. Subik K, Lee JF, Baxter L, Strzepek T, Costello D, Crowley P, Xing L, Hung MC, Bonfiglio T, Hicks DG, Tang P. The Expression Patterns of ER, PR, HER2, CK5/6, EGFR, Ki-67 and AR by Immunohistochemical Analysis in Breast Cancer Cell Lines. Breast Cancer (Auckl). 2010; 4:35–41.
4. Holliday DL, Speirs V. Choosing the right cell line for breast cancer research. Breast Cancer Res. 2011; 13:215.
5. Hu X, Stern HM, Ge L, O'Brien C, Haydu L, Honchell CD, Haverty PM, Peters BA, Wu TD, Amler LC, Chant J, Stokoe D, Lackner MR, et al. Genetic alterations and oncogenic pathways associated with breast cancer subtypes. Mol Cancer Res. 2009; 7:511–22.
6. Kao J, Salari K, Bocanegra M, Choi YL, Girard L, Gandhi J, Kwei KA, Hernandez-Boussard T, Wang P, Gazdar AF, Minna JD, Pollack JR. Molecular Profiling of Breast Cancer Cell Lines Defines Relevant Tumor Models and Provides a Resource for Cancer Gene Discovery. PLoS ONE. 4:e6146
7. Wasielewski M, Elstrodt F, Klijn JG, Berns EM, Schutte M. Thirteen new p53 gene mutants identified among 41 human breast cancer cell lines. Breast Cancer Res Treat. 2006; 99:97–101.
8. Neve RM, Chin K, Fridlyand J, Yeh J, Baehner FL, Fevr T, Clark L, Bayani N, Coppe JP, Tong F, Speed T, Spellman PT, DeVries S, et al. A collection of breast cancer cell lines for the study of functionally distinct cancer subtypes. Cancer Cell. 2006; 10:515–527.
